# Tissue Levels of Flurbiprofen in the Rat Plantar Heel after Short-Duration Topical Iontophoresis Are Sufficient to Induce Pharmacodynamic Responses to Local Pain Stimuli

**DOI:** 10.3390/pharmaceutics12070608

**Published:** 2020-06-30

**Authors:** Yilu Cai, Ye Zhou, Peiyan Zhang, Yogeshvar N. Kalia, Tais Gratieri, Yong Chen

**Affiliations:** 1 Laboratory for Drug Delivery & Translational Medicine, School of Pharmacy, Nantong University, 19 Qixiu Road, Nantong 226001, Jiangsu Province, China; cyl_2017@163.com (Y.C.); zy_2018@163.com (Y.Z.); 15205178308@163.com (P.Z.); 2 School of Pharmaceutical Sciences, University of Geneva & University of Lausanne, CMU, 1 rue Michel Servet, 1211 Geneva, Switzerland; yogi.kalia@unige.ch; 3 Laboratory of Food, Drugs and Cosmetics (LTMAC), University of Brasília, Brasília 70910-900, Brazil; tgratieri@unb.br

**Keywords:** flurbiprofen, iontophoresis, plantar heel pain, local delivery

## Abstract

The objective of this study was to investigate the topical iontophoresis of flurbiprofen (FBF) as a means to enhance its local bioavailability and thereby provide an improved and targeted treatment of plantar heel pain. Initial in vitro experiments using porcine ear skin investigated iontophoretic transport of FBF under different conditions. Local FBF biodistribution in the rat paw in vivo was compared after topical or oral administration. Efficacy of pain management was investigated using a plantar incisional model by evaluating pharmacodynamic responses to local pain stimuli. The results demonstrated that iontophoresis of FBF significantly increased cutaneous deposition and transdermal permeation of FBF as compared to passive delivery—it also enabled drug input to be controlled by modulation of current density and drug concentration (r^2^ > 0.99). Topical iontophoresis of FBF in vivo enabled higher drug levels in skin and muscle in rat plantar aspect and superior pharmacodynamic responses to local pain stimuli, in comparison to oral and passive delivery. In conclusion, short-duration topical iontophoresis of FBF may better help to relieve plantar heel pain than oral or passive administration, which should be of clinical interest.

## 1. Introduction

The integration of an electro-medical technique—iontophoresis, which refers to the ordered movement of ions under a potential gradient—into the field of topical and transdermal drug delivery [1], has shown its ability to make the “undeliverable” deliverable (e.g., cetuximab [2]) or to make the suboptimal candidates optimal (e.g., ARN14140 [3]). Moreover, this powerful technique can individualize the administered dose more readily than many other delivery methods—the transdermal drug flux is adjustable by tuning the intensity of the current applied. Furthermore, transdermal iontophoresis is a non-invasive delivery method that exerts its effect through an additional driving force, the potential gradient, which acts on the molecules rather than the skin. Iontophoresis also enables locally enhanced intraepidermal [4], intramuscular [5], intraocular [6], or intratumoral [7] drug delivery with lower levels of systemic drug exposure.

We have recently focused on local delivery of therapeutics into the plantar heel for the management of heel disorders. Plantar heel pain is commonly caused by soft tissue related fat pad atrophy or contusion, plantar fascia rupture, and plantar fasciitis [8], and may severely affect daily activities and mobility. Although the pain sometimes subsides after conservative physical treatments, it can increase after activity and require pharmacotherapy, e.g., oral nonsteroidal anti-inflammatory drugs (NSAIDs) or local injection of corticosteroids [9]. However, chronic administration of NSAIDs is associated with gastrointestinal problems and an increase in the risk of heart attacks [10]. Furthermore, multiple local injections can result in the rupture of the plantar fascia or shrinkage of the fat pad [8]. Therefore, effective local delivery of anti-inflammatory and/or analgesic agents into the pathological soft tissues of the plantar heel would be of significant interest; however, the passive drug flux across the very thick stratum corneum covering the painful plantar heel from conventional creams or patches is unlikely to produce meaningful therapeutic effects. In light of the obstacles for effective passive delivery, iontophoretic administration might provide a potential solution for locally enhanced drug delivery into the plantar heel.

Previous publications towards the application of iontophoresis on plantar heel were rare and not described in detail. Nevertheless, the very limited existed data already opened the door to the treatment of local plantar disorders by using iontophoretic administration. For example, it has been demonstrated in a clinical study that iontophoresis of “0.4% dexamethasone” in conjunction with other traditional modalities for plantar fasciitis provided more immediate pain relief than traditional modalities alone [11]. In another clinical study, 5% acetic acid iontophoresis combined with taping offered relief from stiffness symptoms [12]. As mentioned, the drug levels in local tissues responsible for these clinical cures were not elaborated, and some basic conditions used in these studies were somewhat vague, for example, the composition of “0.4% dexamethasone” was not described.

**Figure 1:**
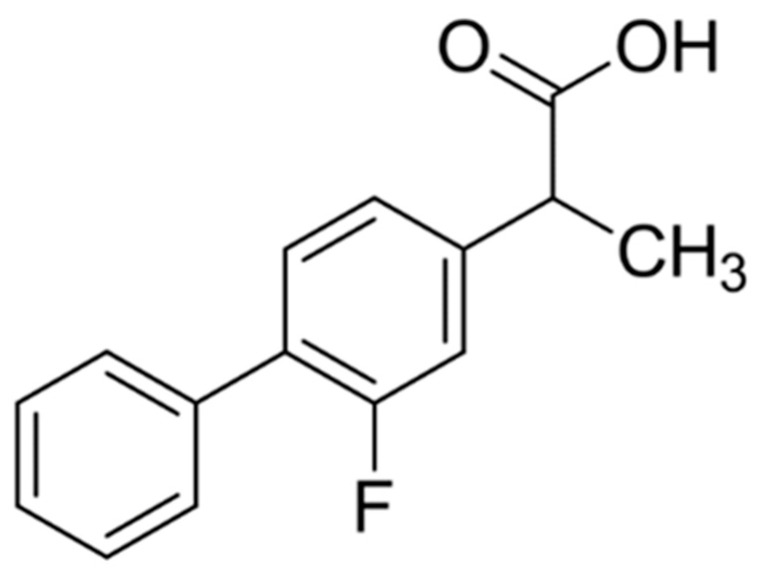
Chemical structure of flurbiprofen (FBF; M.W. 244.33, pKa 4.4).

In this study, the different local drug distributions and pharmacoresponses following passive or iontophoretic delivery of flurbiprofen (FBF, Figure 1) into plantar aspect of rats were compared. Besides oral dosages, FBF is also available via cataplasms, which has been marketed in Asia (Zepolas^®^ in Japan and Debaian^®^ in China), for the management of local pain and soft tissue injuries, given its high potency for the relief of mild to moderate pain accompanied by inflammation (e.g., bursitis, tendinitis, soft-tissue trauma) and appropriate lipophilicity (unionized type) for transdermal delivery. After in vitro experiments using porcine ear skin to determine the optimal iontophoretic parameters, preliminary in vivo studies were performed to determine local drug delivery to the rat paw and compared to oral administration. Finally, in order to investigate the pharmacodynamic responses after receiving topical FBF administration, a validated incisional pain model on rat plantar aspect was established.

Therefore, the specific aims of this study were (i) to evaluate the stability of FBF exposed to porcine skin and current, (ii) to evaluate the impact of different parameters on iontophoretic delivery of FBF across porcine skin in vitro, (iii) to determine FBF distribution in rat plantar skin and muscle after short-duration topical iontophoresis in vivo, and (iv) to compare the efficacy of pain management following different FBF administration regimens by using a validated incisional pain model.

## 2. Materials and Methods

### 2.1. Materials

Flurbiprofen (FBF) was purchased from Hubei Widely Chemical Technology (Wuhan, China). 4-(2-hydroxyethyl)-1-piperazineethanesulfonic acid) (HEPES), hydroxyethyl cellulose (HEC), silver wire, and silver chloride were purchased from Sigma-Aldrich (Shanghai, China). PEG 400 and phosphoric acid was bought from Richjoint Chemical Reagents (Shanghai, China). PVC tubing (3 mm ID, 5 mm OD) used to prepare salt bridge assemblies was obtained from Daoguan Rubber & Plastic (Shanghai, China). Agarose was obtained from Saiguo Biotech (Guangzhou, China). Pentobarbital sodium was supplied by Shandong Xiya Reagent (Linyi, China). Inhalable anesthetic isoflurane was purchased from Yuyan Biotech (Shanghai, China). HPLC-grade acetonitrile and methanol were supplied by Merck KGaA (Darmstadt, Germany). All aqueous solutions were prepared using Milli-Q^®^ water (resistivity > 18 MΩ.cm). All other chemicals were at least of analytical grade.

### 2.2. Skin Source

It is difficult to obtain human heel skin for in vitro iontophoretic transport studies, and there have been no validated surrogates since conventional animal skins were not able to precisely reflect the dry, thick, and compact texture of its stratum corneum covering the human heel skin. Nevertheless, the porcine skin, a good model for normal human skin, was used to roughly estimate the effect of iontophoretic conditions on FBF transport kinetics since it is recognized as a good model for human skin [13,14]. Porcine ears were supplied from a local abattoir (Yongxing; Nantong, China) within a few hours of sacrifice and were cleaned under cold running water. The skin was removed carefully from the outer region of the ear and separated from the underlying cartilage with a scalpel. The skin was then punched out into round disks (diameter ~30 mm), dermatomed (thickness ~1 mm) with a slicer assembly (Thomas Scientific; Swedesboro, NJ, USA), wrapped in Parafilm^TM^ and stored at −20 °C for a maximum period of 1 month. Prior to use, the skin samples were thawed at room temperature for a period of 10 min and excess hair was trimmed with clippers.

### 2.3. Animals

Male Sprague-Dawley rats (SD rats, B.W. 220 ± 20 g) were used for local biodistribution and pharmacodynamic studies. They were supplied by the Laboratory Animal Center of Nantong University. All the animals were in a 12 h dark-light cycle animal facility with controlled temperature and humidity and had free access to regular chow and water except the test sessions. All animal experimental protocols were approved by the Institutional Animal Care and Use Committee, Nantong University (NTU-19030663-6; 06 March 2019), and were carried out in accordance with the “NIH Guide for the Care and Use of Laboratory Animals”.

### 2.4. Stability Studies

To determine the stability of FBF in the presence of skin, 1 mL of FBF solution (4.88 mg/mL, in 20 mM HEPES; pH 8.3) was placed in contact with the epidermal or dermal porcine skin surfaces, respectively, for 12 h. Aliquots (0.1 mL) was taken from the drug solution followed by appropriate dilutions, and the remaining percentage of FBF was measured by using HPLC-UV. To determine the stability of FBF in the presence of and electrical current, 2 mL of FBF solution (4.88 mg/mL, in 20 mM HEPES; pH 8.3) was subjected to a current of 1 mA for 8 h. Salt bridges ensured connectivity between the cathode and the formulation. Aliquots (0.1 mL) were taken from the drug solution followed by appropriate dilutions, and the remaining FBF was measured by using HPLC-UV.

### 2.5. Iontophoretic Transport In Vitro

#### 2.5.1. Experimental Set-Up

Iontophoretic transport studies were conducted to quantify skin deposition and cumulative permeation of FBF. The dermatomed porcine skin was clamped in vertical Franz diffusion cells (Kehua Glassware Instrument; Nantong, China) and allowed to equilibrate for 30 min before the transport experiments. The area of skin exposed to the donor formulation was ~2.0 cm^2^. The donor compartment was filled with 1 mL of drug solution (dissolved in 20 mM HEPES; pH 8.3) and was connected to the cathode (AgCl) via a salt bridge assembly (3% agarose in 0.1 M NaCl). The receiver compartment was used as the anode (Ag) and was filled with 12 mL of PBS solution (pH 7.4, mixed with 20% PEG 400, *v*/*v*). The receiver solution was continuously stirred, and the temperature was maintained at 32 °C using a dynamic water bath system. FBF solution was pipetted into each donor compartment, and current application was initiated using a direct power supply (Kepco^®^ APH 1000 M; Flushing, NJ, USA). During the experiment, samples (1 mL) were withdrawn from the receiver compartment hourly for 8 h and replaced with fresh receiver fluid. Upon completion of the experiments, the diffusion cells were dismantled and the skin was washed with running water to remove any residual formulation on the surface. The formulation-exposed area was cut into small pieces and extracted for 12 h with the mobile phase used for HPLC analysis (See Section 2.8). The skin extraction medium and the samples from the receiver compartment were centrifuged at 12,000 rpm for 15 min. The supernatant was filtered through a nylon filter membrane with a pore size of 0.22 µm. All samples were then analyzed by HPLC-UV.

#### 2.5.2. Effect of Current Density

The effect of the current density (0.1, 0.3, and 0.5 mA/cm^2^) on FBF transport was investigated. 4.88 mg/mL (i.e., 20 mM) of FBF solution was applied in the donor compartment and the experimental set-up was as described above. Passive transport experiments using the same set-up but in the absence of current served as the control (current = 0 mA/cm^2^).

#### 2.5.3. Effect of Concentration

The effect of drug concentration (1.22, 2.44 and 4.88 mg/mL) on FBF transport was investigated. The current was fixed at 0.5 mA/cm^2^ and the experimental set-up was as described above.

#### 2.5.4. Effect of Formulation

Hydrogels have been extensively used as drug reservoirs in iontophoretic patches due to the ease of use [4]. In this study, HEC hydrogel was prepared to incorporate FBF. Being a simple and cheap formulation with good electrical conductance, HEC hydrogel was also used in some previously reported iontophoretic studies [5,15]. Briefly, HEC powder was slowly dispersed in HEPES solution (20 mM, pH 8.3) at room temperature. After swelling for 30 min, mild agitation was initiated until the polymeric solution became transparent. FBF was then added to the solution. Two preservatives, methylparaben and propylparaben, dissolved in minimum quantity of ethanol, were also added. The pH of the mixture was reconfirmed by using a pH meter. A transparent, viscous HEC hydrogel was formed. The composition (*w*/*w*) of the hydrogel was 0.488% FBF, 5% HEC, 0.26% methylparaben, and 0.03% propylparaben. FBF delivery following iontophoresis for 8 h at 0.5 mA/cm^2^ from FBF hydrogel (1 g, 0.488% *w*/*w*) was compared with that from FBF aqueous solution (1 mL, 4.88 mg/mL) under the same conditions.

#### 2.5.5. Effect of the Duration of Current Application

Iontophoretic FBF delivery for different durations (0.5 h, 1 h, 2 h, 4 h, and 8 h) at 0.5 mA/cm^2^ from the FBF hydrogel (0.488%, *w*/*w*) was evaluated. Upon completion of the experiments at each predetermined time point, the assembly was dismantled, and the FBF amounts in skin and in receiver compartment were then analyzed.

### 2.6. Biodistribution Studies In Vivo

Prior to the experiments, the hind paws of the rats were carefully examined to ensure the integrity of the plantar surface. Then, the rats were anaesthetized with pentobarbital sodium (30 mg/kg, i.p.), and the plantar skin of the right hind paw was cleaned with a piece of cotton soaked in warm water. An electrode tape patch (Wandom Medical Devices; Guilin, China) was cut into a rectangle with an area of 1 cm × 2 cm. After peeling off the release liner, 0.1 g of 0.6% (*w*/*w*) FBF hydrogel, prepared according to the same procedure as described in Section 2.5.4, was coated evenly on the exposed surface of the adhesive matrix. The drug-loaded side of the patch was fixed tightly onto the plantar aspect of the right hind paw from the end of the heel and extending towards the toes with the help of adhesive films, and the metal electrode in the opposite side of the patch was connected to the cathode lead of the APH 1000 M power generator. A blank electrode tape patch, connected to the anode lead, was applied on the skin of the left hind leg, serving as the return electrode. A constant current (0.5 mA/cm^2^) was delivered for 0.5 h. A comparative study investigating passive permeation for 30 min was also performed using the same assembly as described above but without current application. Upon completion of iontophoretic or passive delivery, the patch and the residual formulation on the plantar aspect of the right hind paw were removed, and the blood, skin, and muscle samples were taken immediately (Ionto 0.5 h or Passive 0.5 h), or after an additional 1 h (Ionto 0.5 h + 1 h or Passive 0.5 h + 1 h) or 2 h (Ionto 0.5 h + 2 h or Passive 0.5 h + 2 h) to allow the drug to distribute in vivo.

A blood sample (0.5 mL) was withdrawn by cardiac puncture and the plasma was isolated by centrifugation (5000 rpm) and stored at −80 °C. The hind paws were immediately frozen in liquid nitrogen in order to retard possible drug partition between the skin and the muscle beyond the preset time window. A portion of the frozen plantar skin with the underlying muscle (area, ~0.5 cm × 0.5 cm; total thickness, ~3 mm), which was localized at the area facing to the center of the patch, was excised from the right hind paw using a scalpel. The skin and the muscle were carefully separated, weighed, and stored at −80 °C for further analysis. Samples from the contralateral paw (left hind paw) were also taken in the same manner in order to investigate the possible redistribution effect.

FBF biodistribution after oral administration was also performed. The rats were fasted for 6 h prior to experiments but water was provided ad libitum. Each rat was administered 0.5 mL of FBF aqueous suspension (0.12% FBF, *w/v*) intragastrically, and then anaesthetized with pentobarbital sodium (30 mg/kg, i.p.) in order to keep the general hemodynamics similar as that in the anaesthetized rats in the groups receiving topical administration. The blood, skin, and muscle samples were then taken after 0.5 h (Oral 0.5 h), 1.5 h (Oral 1.5 h), or 2.5 h (Oral 2.5 h) in the same manner as described above.

Prior to analysis, 200 μL of the freshly thawed plasma sample was mixed with 200 μL of HPLC-grade acetonitrile on a vortexer and then centrifuged at 12,000 rpm for 10 min to collect the supernatant, which was then subjected to HPLC analysis. The skin and muscle samples were cut into small pieces and soaked in 5 mL of the mobile phase used in HPLC analysis. The mixture was homogenized by using a homogenizer (Bio-Gen PRO 200, PRO Scientific Inc.; Oxford, CT, USA) for 1 min, followed by stirring on a magnetic stirrer (300 rpm) for 12 h at room temperature. The mixtures were then filtered through a membrane filter (pore size 0.45 μm). The filtrates were lyophilized and subsequently reconstituted using 200 μL mobile phase. The mixtures were then centrifuged (12,000 rpm, 5 min) and the supernatants were subjected to HPLC-UV analysis. The extraction methods were validated (See Section 2.8).

### 2.7. Pharmacodynamic Studies

#### 2.7.1. Drug Administration

The efficacy of pain management was investigated using an animal incisional model described previously [16], in which an incision on the plantar aspect of the rat hind paw was made after drug administration, followed by quantitative evaluations of the pain responses to mechanical or thermal stimuli. The rats were grouped according to different regimens of drug administration: (a) G0, no treatment; (b) G1, topical application of blank hydrogel + iontophoresis; (c) G2, topical application of FBF hydrogel + iontophoresis; (d) G3, topical application of a marketed FBF hydrogel patch (cataplasms) and (e) G4, oral administration of FBF suspension. For G1–G3 groups, the rats were fixed by cylindrical plastic fixator with the hind legs stretched out from the slots and fixed by adhesive films in order to facilitate topical drug administration. All animals in this study were awake before the induction of hind paw surgery.

The rats from G0 did not receive any treatment or surgery. For G1 and G2 groups, the same iontophoretic assembly used in the investigation for FBF biodistribution was used, and 0.1 g of blank hydrogel (5% HEC in saline) or 0.1 g of 0.6% (*w*/*w*) FBF hydrogel were coated evenly on the exposed surface of the adhesive matrix, respectively. The current density and duration of current application for these two groups were consistent with the iontophoretic conditions used in the biodistribution studies (0.5 mA/cm^2^, 30 min). For G3 group, a small patch (1 cm × 2 cm), cut from a marketed FBF hydrogel patch (Debaian^®^; strength, 40 mg; area, 13.6 cm × 10.0 cm; Beijing Tide Pharmaceutical Co., Ltd., Beijing, China), was applied directly onto the plantar aspect of the right hind paw for 30 min, followed by the same cleaning procedure as that in G2. For G1–G3 groups, upon completion of the topical administration, the patches were removed and the skin surface of the plantar aspect of the right hind paw was cleaned by cotton swabs; then, the rats were dismantled from the cylindrical plastic fixator, followed immediately by the induction of hind paw surgery. For G4 group, the rats were administered with 0.5 mL of FBF suspension (0.12% FBF, *w/v*) intragastrically and returned to their cages, and the surgery was initiated after 30 min. It is necessary to emphasize that all of the rats from G2–G4 received the same dose of FBF (~0.6 mg) and experienced the same duration from the initiation of saline/drug administration to the initiation of the surgery (~30 min).

#### 2.7.2. Induction of Hind Paw Surgery

Briefly, a rat taken from G1–G4 was placed in a transparent acrylic box and anaesthetized with 3.5% isoflurane mixed with oxygen at a flow rate of 3.5 L/min. A 1-cm longitudinal incision was made through skin, fascia, and muscle of the plantar aspect of the right hind paw, beginning 0.5 cm from the end of the heel and extending towards the toes. The plantaris muscle was elevated using forceps and incised longitudinally. Gentle pressure with a piece of sterilized cotton was applied for hemostasis, and the wound was sutured with two single stitches using 5-0 nylon. Upon completion of the surgery, which took about 5 min, the rat was left in the cage until totally recovered from the anesthetic, which took another 10 min, followed by measuring the pain responses, respectively. It should be pointed out that all of the rats from G1–G4 experienced the same duration from the initiation of the surgery to the first time point for the pain evaluation by Von Frey test or Hargreaves test (~15 min = 5 min of surgery + 10 min of awakening).

#### 2.7.3. Von Frey Test

The mechanical hyperalgesia was measured by testing the paw withdrawal threshold (PWT) to mechanical stimuli using von Frey filaments (Stoelting; Wood Dale, IL, USA) [17,18]. The PWT was defined as the lowest force that caused at least two withdrawals of the injured paw in three trials. Briefly, experimental rats were placed in custom-made individual transparent cubicles on elevated metal mesh floor right after the surgery, as described above. The hind-paw plantar surface was stimulated with a series of von Frey filaments with the bending forces equivalent to 4, 6, 8, 10, 15, and 26 g. Filaments were introduced through the wire mesh and applied perpendicularly to the surrounding surgical area from the least to the greatest forces. The responses of the injured paw (withdrawal or not) were recorded. If there was no response to the force of 26 g, the tests were stopped to avoid further injury to the paw. The tests were performed at 15, 30, 60, and 120 min after the initiation of the surgery.

#### 2.7.4. Hargreaves Test

The thermal hyperalgesia was assessed by recording the paw withdrawal latency (PWL) to radiant heat using Hargreaves radiate heat apparatus (Model 390, IITC Life Science; Woodland Hills, CA, USA) [19]. The PWL was defined as the time interval from the radiant giving to the paw lift. Briefly, experimental rats were placed on a glass plate and a movable radiant was placed underneath the glass floor, which was able to deliver radiant heat to the surgical area. Three tests were conducted within a 5-min period and the average value was calculated. Before the surgery, the radiant heat intensity was adjusted so that baseline PWL was between 12 and 15 s. The tests were performed at 15, 30, 60, and 120 min after the initiation of the surgery.

### 2.8. Analytical Methods for Quantification of FBF

Quantification of FBF was conducted by using a HPLC-UV system consisted of a LC-10AT VP pump, a SPD-10A VP UV-VIS detector, a CTO-10A column oven, a SIL-10AF auto sampler and a SCL-10A VP controller (Shimadzu Corporation; Kyoto, Japan). Data were collected and processed using LC-solution software. Isocratic elution was performed on a Diamonsil C18 column (150 mm × 4.6 mm I.D., 5 μm), and an EasyGuard C18 guard column (10 × 4.0 mm I.D., 5 μm) was mounted upstream from the analytical column (Dikma Technologies; Beijing, China). The column temperature was kept at 35 °C, the flow rate was maintained at 1.0 mL/min, and the UV absorbance wavelength was set at 254 nm. The injection volume was 20 μL. The mobile phase comprised 23% (*v*/*v*) phosphate buffer (20 mM NaH_2_PO_4_; pH 3.0) and 77% (*v*/*v*) methanol. Different concentrations of FBF was spiked in blank porcine skin extract solution, rat plasma, and rat muscle extract solution, and were quantified for method validation. The results showed good precision and accuracy for both intra-and inter-day analyses, as shown in Table 1. The specificity of the analytical method was verified with respect to endogenous compounds present in different tissue samples. The calibration range was 50–5000 ng/mL. The limit of quantification (LOQ) was 50 ng/mL.

The methods to extract FBF from skin and muscle were validated by spiking the tissue samples with three different known amounts of drug solution. Briefly, 50 μL of FBF methanol solution (2, 1, or 0.2 mg/mL, respectively) was added either onto the dermal surface of a piece of freshly dermatomed porcine skin (diameter 1.6 cm) or into a tube which contained approximately 1 g of freshly excised muscle tissue from hind limb of a rat. The samples were dried overnight at room temperature and then subjected to the extraction procedure (See Section 2.6). Similar measures were taken to validate the recovery rate of FBF in plasma samples. Briefly, 50 μL of FBF methanol solution (2, 1 or 0.2 mg/mL, respectively) was mixed with 200 μL of blank rat plasma. The mixture was immediately subjected to extraction procedure (See Section 2.6). The recovery rates of FBF were determined by calculating the ratio of the amount extracted from the skin, muscle, or plasma to the amount added, and the results are shown in Table 2.

### 2.9. Statistical Analysis

Data were expressed as the mean ± SD. Outliers determined using the Dixon test were discarded. Results were evaluated statistically using either analysis of variance (ANOVA) or Student’s *t*-test. Student–Newman–Keuls test was used when necessary as a post-hoc procedure. The level of significance was fixed at *p* < 0.05.

## 3. Results and Discussion

### 3.1. FBF Stability in Presence of Skin and Electric Current

The concentration of FBF in solution after exposure to porcine epidermis and dermis for 12 h were 98.83 ± 2.68% and 91.79 ± 3.73%, respectively, of the initial values. After 8 h of current application, the solution concentration of FBF was 99.4 ± 1.9% of the initial value. The results indicated that FBF was stable under the conditions for the planned iontophoretic delivery experiments.

### 3.2. FBF Transport through Porcine Skin In Vitro

**Figure 2:**
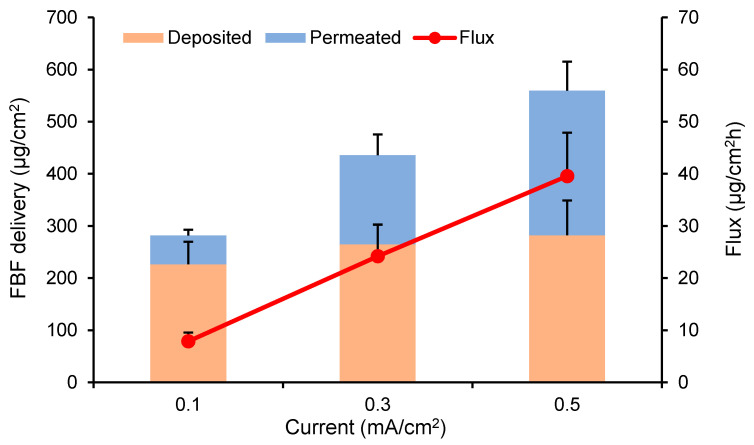
Effect of current density (0.1, 0.3, and 0.5 mA/cm^2^) on FBF delivery and the steady state flux for 8 h of transdermal iontophoresis using a 4.88 mg/mL solution. (Mean ± SD; *n* = 6).

Percutaneous penetration of FBF across dermatomed porcine skin after passive delivery for 8 h was not detected, and only a limited amount of FBF was found after skin extraction (37.02 ± 4.38 μg/cm^2^). The cumulative permeation of FBF after iontophoresis for 8 h at 0.1, 0.3 and 0.5 mA/cm^2^ was 54.84 ± 10.93, 170.77 ± 40.11, and 277.12 ± 55.72 μg/cm^2^, respectively, and the drug fluxes were 7.87 ± 1.70, 24.20 ± 6.06, and 39.56 ± 8.34 μg/cm^2^ h. Increasing current density resulted in statistically significant linear flux enhancements (r^2^ > 0.99). Appreciable amounts of FBF were also found retained within the skin during iontophoresis; however, there was not a statistically significant increase in skin deposition as a function of current density (Figure 2).

**Figure 3:**
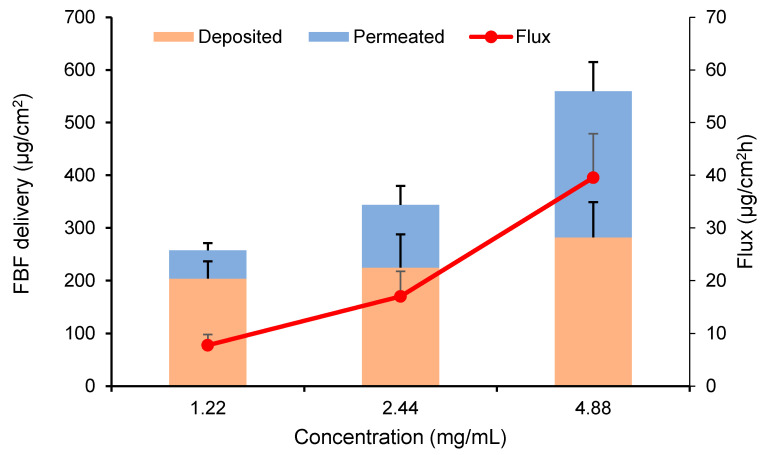
Effect of donor concentration (1.22, 2.44, and 4.88 mg/mL) on FBF delivery and the steady state flux for 8 h of transdermal iontophoresis at 0.5 mA/cm2. (Mean ± SD; *n* = 6).

Cumulative permeation of FBF after iontophoresis for 8 h at 0.5 mA/cm^2^ using drug concentrations of 1.22, 2.44, and 4.88 mg/mL was 53.55 ± 13.52, 119.02 ± 36.10, and 277.12 ± 55.72 μg/cm^2^, respectively. Increasing drug concentrations resulted in linearly increased cumulative amounts and drug fluxes (r^2^ > 0.99). The differences were shown to be statistically significant in the cumulative permeation but not in the skin deposition (Figure 3).

The transport number is the fraction of the total charge carried by a specific ion and expresses its efficiency as a charge carrier [20]. The sum of the transport numbers of all ions presented in the system must add up to 1. Although the use of salt bridges enabled the removal of competing anions in the donor compartment, the cations in the receiver compartment (e.g., Na^+^) were able to electromigrate across the skin towards the donor compartment due to the high electromobility and, therefore, they were principal charge carrier: only < 4% of the total current was carried by anionic FBF (Table 3). Topical iontophoresis enabled fast, directional migration of charged drugs. In this study, iontophoresis enabled 6.6- to 26.8-fold increases in the delivery efficiency, in comparison to passive delivery, and up to ~42% of the drug in the donor was successfully delivered (Table 3).

HEC hydrogel of FBF was prepared in order to facilitate further in vivo studies on local biodistribution and pharmacodynamics following iontophoresis. The gel was prepared in weakly basic HEPES solution (pH 8.3) in order to keep FBF ionized in the matrix. The gel was stable for at least 1 month at room temperature. The cumulative permeation after iontophoresis for 8 h at 0.5 mA/cm^2^ was 242.26 ± 44.85 μg/cm^2^, which was statistically equivalent with that observed from the solution based-FBF (c.f. 277.12 ± 55.72 μg/cm^2^). There was also no statistically significant difference between the steady-state fluxes from FBF aqueous formulation and FBF hydrogel (39.56 ± 8.34 and 33.17 ± 5.84 μg/cm^2^ h, respectively). Although FBF was entrapped in the polymer matrix structure, the iontophoretic transport was obviously not hindered.

**Figure 4:**
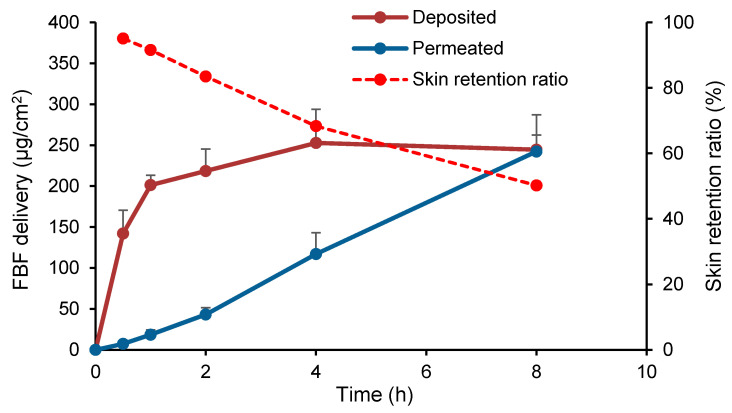
Effect of the duration of current application (0.5, 1, 2, 4, and 8 h) on FBF delivery and skin retention ratio after transdermal iontophoresis at 0.5 mA/cm2 using a 0.6% (*w*/*w*) HEC hydrogel. (Mean ± SD; *n* = 6).

The relative contributions of skin deposition and cumulative permeation to the total iontophoretic delivery of FBF as a function of the iontophoretic duration are shown in Figure 4. While the amount of FBF permeated across the skin gradually increased as a function of time, the amount deposited increased dramatically in the first hour, but almost plateaued subsequently (201.31 ± 11.88, 218.44 ± 26.79, 252.77 ± 41.04, and 244.47 ± 18.06 μg/cm^2^ for 1, 2, 4, and 8 h, respectively, without statistically significant differences), indicating that deposition was a saturable process [5,21]. The skin retention ratio of FBF, given by the ratio of the amounts deposited to the summed amounts of deposited and permeated, also declined with the extension of current application, which confirmed the lack of dependence between the amount of drug retained in the skin and the duration of current application (Figure 4). Although it has been reported that drugs can be bound to epidermis and dermis, in particular lipophilic compounds, the cutaneous binding sites were supposed to be limited since only when the drug concentrations were limited in a certain low range (e.g., 10^−7^ to 10^−3^ mol/L), the binding of drugs to skin was linear and not saturable [22]. Therefore, it was assumed once the rapid delivery of FBF by iontophoresis enabled “full accommodation” in skin, the molecules “entering” into the skin tended to diffuse further into the receiver compartment and, hence, the steady state flux was achieved. Nevertheless, the lipid matrix in the epidermis and certain hydrophilic biopolymers in the dermis provided large capacity to retain FBF (log P > 2.2 in acidic and neutral conditions) [23]. This might help to maintain the drug levels in skin and the very adjacent underlying tissues in a certain time period following a short-duration topical iontophoresis in vivo.

### 3.3. Biodistribution Studies In Vivo

Considering the low transport flux in vitro, it was not unexpected that the passive delivery of FBF across rat plantar skin for 0.5 h in vivo was poor. Only modest amounts of FBF were observed in the treated skin immediately after passive delivery (3.35 ± 0.66 μg/g for Passive 0.5 h) and after an additional 1 h (0.77 ± 0.53 μg/g for Passive 0.5 h + 1 h), whereas the amounts of FBF in the muscle underlying the treated skin, as well as systemic drug exposure, were not detectable (Table 4). Compared to passive delivery, topical short-duration iontophoresis on the plantar aspect of the rats for 0.5 h produced a remarkable improvement in the total bioavailability of FBF, as significantly greater amounts of FBF were retained in the skin from the treated area (46.74 ± 19.22 μg/g for Ionto 0.5 h; *p* < 0.05). Moreover, FBF exposure in muscle underlying the iontophoretic delivery site and in plasma were also measurable (1.87 ± 0.41 μg/g for Ionto 0.5 h), but the concentrations were much lower than that retained in the skin (Table 4).

Although FBF concentrations quantified in the muscle beneath the treated skin area declined with time after iontophoresis (1.87 ± 0.41, 0.93 ± 0.35, and 0.60 ± 0.28 μg/g at Ionto 0.5 h, Ionto 0.5 h + 1 h and Ionto 0.5 h + 2 h, respectively), they were still superior to the concentrations in the muscle from contralateral paw, which were 0.11 ± 0.04, 0.17 ± 0.12 and 0.14 ± 0.06 μg/g at the corresponding sampling points, respectively. In addition, FBF levels in the blood were lower than those in the muscles under the iontophoretic delivery site, but higher than those in the muscles from contralateral paw. These results unequivocally demonstrated that although FBF redistribution from systemic circulation to the muscular tissue does occur, the direct percutaneous penetration during topical iontophoresis and continuous drug release from the “skin depot” is predominantly responsible for creating and maintaining comparatively higher levels of FBF in the muscle under the iontophoretic delivery site.

These results might also suggest that the local microenvironment in skin and underlying soft tissue of the plantar aspect displayed the “drug-reservoir” effect more functionally than those in the limbs. It was reported in the previous studies that, although high levels of drug were observed in the muscle/knee joint underlying the iontophoretic site, they declined rapidly after termination of iontophoresis by loss to the local blood flow and tended to be equivalent with those in the untreated counterparts [5]; however, in this study, the FBF levels in the muscle under the treated site at Ionto 0.5 h + 1 h and Ionto 0.5 h + 2 h (0.93 ± 0.35 and 0.60 ± 0.28 μg/g, respectively) were still statistically greater than those in the muscle from the contralateral paw at corresponding time points (c.f. 0.17 ± 0.12 and 0.14 ± 0.06 μg/g, respectively). The detailed mechanism behind this phenomenon needs to be clarified in future studies; however, the unique morphology and composition of plantar skin, with its much thicker and lipid-richer stratum corneum [24], might contribute to the high capacity of drug retention. Moreover, the subcutaneous fat pads, present in the human sole [25] and in rat paws [26], might also be helpful in sustaining the drug release. These lipid-rich structures, though evolved to tolerate routine surface pressures, might help to “store” lipophilic compounds, like FBF. The drug resided in these lipid-rich structures of the plantar tissues might subsequently diffuse into the adjacent muscles or local vascular system in a sustainable manner.

In order to understand more clearly about the redistribution effect, FBF concentrations in skin, muscle, and plasma were also measured after oral administration. The orally absorbed FBF must be distributed systemically and then brought into the targeted skin and subcutaneous muscle. The results showed that the resulting systemic exposure after oral administration was much higher than what was observed with topical FBF iontophoresis (Table 5); however, this superior systemic exposure did not translate to higher FBF levels in the plantar muscle and skin. The amounts of FBF distributed into the muscle after oral administration were still significantly lower than those after iontophoresis at 0.5 h and 1 h after the initiation of drug administration: 0.13 ± 0.05 μg/g for Oral 0.5 h vs. 1.87 ± 0.41 μg/g at Ionto 0.5 h, and 0.28 ± 0.11 μg/g for Oral 1.5 h vs. 0.93 ± 0.35 μg/g at Ionto 0.5 h + 1 h. Nevertheless, tissue concentrations represented a larger proportion of systemic exposure than the levels detected in plasma [27]. Given the high plasma protein binding rate (> 99%) [28] and relatively low apparent volume of distribution (0.12 L/kg in human [29], and about 0.11 ~ 0.16 L/kg in rats [30]), orally administered FBF is unlikely to enable fast drug distribution in local superficial tissues within short time.

IC_50_ values of FBF (50% inhibitory concentration for COX-2 inhibition) have been reported to be in a range of 0.01–0.9 μM [31,32], which correspond to concentrations between 2.24 ng/mL and 219.84 ng/mL. In the present study, FBF levels in skin and muscle immediately after a 30-min topical iontophoresis was approximately 46.74 and 1.87 μg/mL, respectively, roughly estimating the mass density of the rat plantar skin and muscle was 1.0 g/cm^3^, which were much greater than IC_50_ of FBF inhibiting COX-2. The drug levels there were still higher than IC_50_ value even after an additional 2 h (32.51 and 0.60 μg/mL for skin and muscle, respectively). Given these data, the FBF levels in plantar skin and muscle within a time window of at least 2 h after a 0.5 h iontophoresis should be able to exert anti-inflammatory and analgesic activities by overwhelmingly inhibiting COX enzymes.

### 3.4. Pharmacodynamics Studies

It was decided to administer the drug before the establishment of the incisional pain model. This was based on the consideration that the integrity of the stratum corneum on the plantar aspect should avoid impairments before application of any drug formulations. This “pre-emptive” analgesic administration does not completely reflect the clinical scenarios of patients with plantar heel pain; however, the investigation in this study if the drug levels measured in local plantar tissues were able to exert any pharmacoresponses to local pain stimuli was crucial to evaluate the “suitability” of this drug delivery system for the management of plantar heel pain.

**Figure 5:**
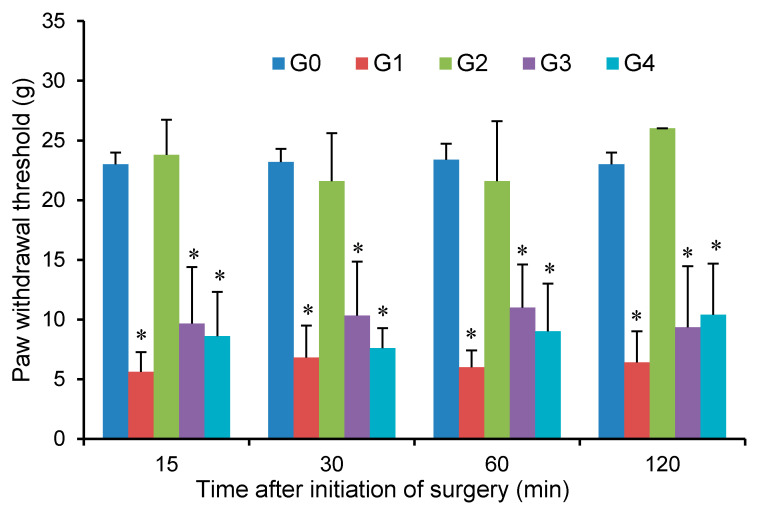
Mechanical pain sensitivity indicating by paw withdrawal threshold (PWT, Von Frey test) among different groups. G0, no treatment; G1, topical application of blank hydrogel + iontophoresis; G2, topical application of FBF hydrogel + iontophoresis; G3, topical application of a marketed FBF hydrogel patch (cataplasms) and G4, oral administration of FBF suspension. *n* = 6; * *P* < 0.05 compared with the baseline of G0 group.

**Figure 6:**
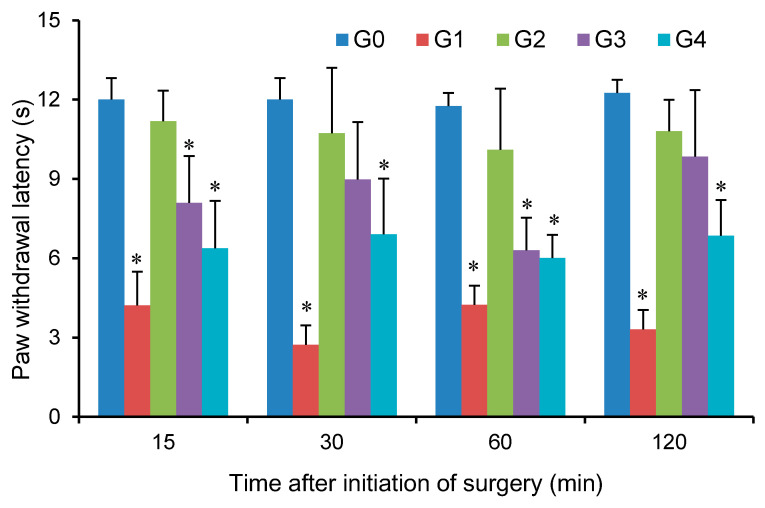
Thermal pain sensitivity indicating by paw withdrawal latency (PWL, Hargreaves test) among different groups. G0, no treatment; G1, topical application of blank hydrogel + iontophoresis; G2, topical application of FBF hydrogel + iontophoresis; G3, topical application of a marketed FBF hydrogel patch (cataplasms) and G4, oral administration of FBF suspension. *n* = 6; * *P* < 0.05 compared with the baseline of G0 group.

In G0 group, the PWT or PWL were tested by applying mechanical or thermal stimuli to the right paws of healthy rats, respectively, which did not differ significantly among the 4 time points for each test (Figure 5 and Figure 6)—these were actually the baseline PWT and PWL. In G1 group, the PWT and PWL were significantly reduced (*p* < 0.01) compared with those of G0 group, indicating that the incisional model for evaluation of pain responses was successfully established. The high sensitivity to the mechanical or thermal stimuli also suggested no effect of blank hydrogel and iontophoresis on plantar pain management.

In G2 group, PWT was not significantly different at 15, 30, 60, and 120 min, but were significantly higher than that of G1 group. The PWL in G2 group was also superior to that of G1 group without any significant decline during the pain evaluation (no statistically significant difference was observed). These results clearly demonstrated that short-duration topical FBF iontophoresis enabled efficacious pain management on the plantar surgical incision during the period of pain evaluation. In G2 group, no statistically significant differences were found for either PWT or PWL when compared to the corresponding value in G0 group, except a single time point when evaluating PWT and PWL at 120 min, where PWT in G2 was slightly higher than that in G0 but PWL was opposite. These were more likely to be attributed to the random error in observations—these need to be further clarified using higher number of animals.

In G3 group, the application of the marketed FBF hydrogel patch provided slightly higher PWT than those of G1 group, but the value between the two groups were not statistically different. However, it was found that the PWL after application of the patch were superior to those of G1 group, suggesting that the patch enabled only modest efficacy of pain management on plantar aspect. The efficacy of the marketed patch to prevent incisional pain arisen from different stimuli was inconsistent—its performance with respect to PWL seems better than for PWT. The exact mechanism behind this difference was impossible to fully understand in this specific study; however, it is noteworthy that the pathophysiological fundaments (e.g., signal transducer) of the body responses to mechanical or radiant heat was totally different [33]. This may translate to the different tolerance to different types of stimuli upon receiving absolutely the same pretreatment of the marketed FBF patch.

In G4 group, the changes of PWT or PWL during the pain evaluation were insignificant, and the values were lower than the corresponding values of the G2 group, demonstrating that this oral dose of FBF was not able to provide rapid pain management on the local tissue injury. This may be explained from the pharmacokinetic properties of FBF after oral administration: the duration between the initiation of oral drug administration and the initiation of the tests (~45 min) was not long enough for FBF absorption by the rat intestine and the subsequent distribution into the local superficial tissues, considering its relatively high plasma *t*_max_ (~3.7 h in rats) [34] and low V_d_ at steady state (0.11–0.16 L/kg in rats) [30]. This was also consistent with the observations in the biodistribution studies where the tissue levels of FBF after oral administration were inferior to those after topical iontophoresis.

### 3.5. Clinical Relevance

Although there is a huge unmet demand for therapies to control plantar heel pain, there has been no clinically validated, well-accepted topical medicinal products developed and marketed. The formidable barrier property of the stratum corneum covering plantar heel effectively prevents efficacious drug delivery from conventional topical formulations. Short-duration topical iontophoresis of analgesics using an “active” patch with acceptable size (e.g., 3 × 3 cm^2^) and current provision (e.g., 0.5 mA/cm^2^) might hopefully overcome this biological barrier and deliver supra-therapeutic levels of drug into the local plantar tissues. Furthermore, some pre-treatments before application of iontophoresis, e.g., foot bathing for sufficient hydration of stratum corneum and/or mechanical exfoliants using a foot file might also be helpful to enhance drug penetration. The last but not the least, topical administration of FBF using short-duration iontophoresis avoids systemic side effects commonly accompanied with oral dosages of NSAIDs; in the meantime, unlike many anesthetics (e.g., lidocaine) which cause transient loss of nerve transmission, there was no evidence that NSAIDs could impair the physical sensation—this would be important to locomotive system. Nevertheless, due to the pathological differences of plantar heel pain among patients undergone various pathogenic experiences, the efficacy to use this method to manage plantar heel pain still needs to be further investigated in clinical trials.

## 4. Conclusions

The results in this study confirm that cathodal iontophoresis remarkably increased topical delivery of FBF, and the electrotransport of FBF was linearly dependent on current density and drug concentration. Topical iontophoresis on rat plantar aspect using a FBF hydrogel enabled much higher amounts of drug deposition in plantar skin and muscle under the treatment site than those after passive or oral FBF delivery, and the unique structures in plantar heel may display a “drug reservoir” function for sustaining the drug release. The improved local drug accumulation also translated to efficacious local pain management on an incisional pain model upon receiving mechanical or thermal stimuli. In summary, short-duration topical iontophoresis of FBF may better help to relieve plantar heel pain than oral or passive administration, which should be of clinical interest.

## Figures and Tables

**Table 1:** Recovery of flurbiprofen (FBF) from porcine skin, rat muscle and rat plasma (*n* = 3).

Theoretical Added Amount (µg)	Skin		Muscle		Plasma	
	Measured Amount (µg)	Recovery (%)	Measured Amount (µg)	Recovery (%)	Measured Amount (µg)	Recovery (%)
10	9.76 ± 0.35	97.60 ± 3.52	10.13 ± 0.16	101.26 ± 1.60	9.29 ± 0.12	92.92 ± 1.17
50	41.41 ± 1.51	82.83 ± 3.02	41.33 ± 0.66	82.67 ± 1.33	44.49 ± 1.74	88.99 ± 3.47
100	81.95 ± 2.38	81.95 ± 2.38	82.41 ± 2.80	82.41 ± 2.80	88.90 ± 3.15	88.90 ± 3.15

**Table 2:** Precision and accuracy values for the analytical method used to quantify FBF in porcine skin, rat muscle and rat plasma (*n* = 3).

	Skin Extract Solution			Muscle Extract Solution			Plasma		
Theoretical Concentration ^a^	5	50	100	5	25	50	100	500	1000
Intra-day assay									
Measured concentration	5.06 ± 0.03	50.64 ± 0.53	101.13 ± 0.71	5.03 ± 0.05	25.30 ± 0.13	48.36 ± 0.11	103.19 ± 3.76	492.98 ± 12.36	974.02 ± 4.66
Precision ^b^	0.50	1.04	0.70	0.91	0.52	0.23	3.64	2.51	0.48
Accuracy ^c^	101.14	101.28	101.13	100.54	101.19	96.72	103.19	98.60	97.40
Inter-day assay									
Measured concentration	5.01 ± 0.06	50.37 ± 0.52	100.03 ± 0.66	5.03 ± 0.04	25.12 ± 0.43	48.65 ± 0.48	102.90 ± 3.51	488.21 ± 14.08	979.29 ± 7.93
Precision ^b^	1.17	1.03	0.66	0.85	1.69	0.98	3.41	2.88	0.81
Accuracy ^c^	100.59	100.07	100.69	100.60	100.48	97.30	102.90	97.64	97.93

^a^ The concentration units of FBF in skin extract solution, muscle extract solution and plasma were μg/mL, μg/mL and ng/mL, respectively. ^b^ Precision = (SD/mean) × 100. ^c^ Accuracy = (obtained concentration/theoretical concentration) × 100.

**Table 3:** Transport flux, transport efficiency and delivery efficiency under different conditions (*n* = 6).

Current (mA)	Concentration (mg/mL)	Total Delivery (μg/cm^2^) ^a^	Total Charge (C) ^b^	Transport Efficiency (%) ^c^	Delivery Efficiency (%) ^d^
0	4.88	37.02 ± 4.38	/	/	1.52
0.2	4.88	281.70 ± 55.15	5.77	3.86	11.55
0.6	4.48	435.60 ± 79.56	17.31	1.99	17.85
1.0	4.88	559.42 ± 123.88	28.85	1.53	22.93
1.0	2.44	343.95 ± 100.12	28.85	0.94	28.19
1.0	1.22	257.58 ± 47.89	28.85	0.70	42.23

^a^ Total delivery = the amount of drug deposited in skin + the amount drug permeated across skin. ^b^ Total charge (C) = the current passed (A) × the duration of current application (s). ^c^ Transport efficiency = transport number × 100%. ^d^ Delivery efficiency = total mean delivery of the drug amount/initial drug amount in donor × 100%.

**Table 4:** FBF biodistribution after topical delivery (*n* = 5 or 6).

Tissue	Sampling Site	FBF Concentration ^a^					
		Ionto 0.5 h	Passive 0.5 h	Ionto 0.5 h + 1 h	Passive 0.5 h + 1 h	Ionto 0.5 h + 2 h	Passive 0.5 h + 2 h
Skin	Treated area	46.74 ± 19.22	3.35 ± 0.66	38.19 ± 17.90	0.77 ± 0.53	32.51 ± 18.37	<LOQ
	Contralateral area	<LOQ ^b^	<LOQ	<LOQ	<LOQ	0.10 ± 0.04	<LOQ
Muscle	Treated area	1.87 ± 0.41	<LOQ	0.93 ± 0.35	<LOQ	0.60 ± 0.28	<LOQ
	Contralateral area	0.11 ± 0.04	<LOQ	0.17 ± 0.12	<LOQ	0.14 ± 0.06	<LOQ
Plasma	Cardiac puncture	0.89 ± 0.42	<LOQ	0.44 ± 0.24	<LOQ	0.18 ± 0.05	<LOQ

^a^ The concentration units of FBF in the skin, muscle and plasma were μg/g, μg/g, and μg/mL, respectively. ^b.^ LOQ: Limit of quantification.

**Table 5:** FBF biodistribution after oral delivery (*n* = 6).

Tissue	Sampling Site	FBF Concentration ^a^		
		Oral 0.5 h	Oral 1.5 h	Oral 2.5 h
Skin	Right hind paw	< LOQ ^b^	< LOQ	0.20 ± 0.04
Muscle	Right hind paw	0.13 ± 0.05	0.28 ± 0.11	0.57 ± 0.15
Plasma	Cardiac puncture	3.38 ± 0.44	5.17 ± 1.23	6.65 ± 1.31

^a^ The concentration units of FBF in the skin, muscle and plasma were μg/g, μg/g and μg/mL, respectively. ^b^ LOQ: Limit of quantification.

## References

[1] Kalia Y.N., Naik A., Garrison J., Guy R.H. (2004). Iontophoretic drug delivery. Adv. Drug Deliv. Rev..

[2] Lapteva M., Sallam M.A., Goyon A., Guillarme D., Veuthey J.L., Kalia Y.N. (2020). Non-invasive targeted iontophoretic delivery of cetuximab to skin. Expert Opin. Drug Deliv..

[3] Singhal M., Merino V., Rosini M., Cavall A., Kalia Y.N. (2019). Controlled iontophoretic delivery in vitro and in vivo of ARN14140—A multitarget compound for Alzheimer’s disease. Mol. Pharm..

[4] Chen Y., Zahui T., Alberti I., Kalia Y.N. (2016). Cutaneous biodistribution of ionizable, biolabile aciclovir prodrugs after short duration topical iontophoresis: Targeted intraepidermal drug delivery. Eur. J. Pharm. Biopharm..

[5] Gratieri T., Pujol-Bello E., Gelfuso G.M., Souza J.G.D., Kalia Y.N. (2014). Iontophoretic transport kinetics of ketorolac in vitro and in vivo: Demonstrating local enhanced topical drug delivery to muscle. Eur. J. Pharm. Biopharm..

[6] Chen Y., Kalia Y.N. (2018). Short-duration ocular iontophoresis of ionizable aciclovir prodrugs: A new approach to treat herpes simplex infections in the anterior and posterior segments of the eye. Int. J. Pharm..

[7] Byrne J.D., Jajja M.R.N., O’Neill A.T., Bickford L., Keeler A.W., Hyder N., Wagner K., Deal A.M., Little R.E., Moffitt R.A. (2015). Local iontophoretic administration of cytotoxic therapies to solid tumors. Sci. Transl. Med..

[8] Tatli Y.Z., Kapasi S. (2009). The real risks of steroid injection for plantar fasciitis, with a review of conservative therapies. Curr. Rev. Musculoskelet. Med..

[9] Goff J.D., Crawford R. (2011). Diagnosis and treatment of plantar fasciitis. Am. Fam. Physician.

[10] Bacchi S., Palumbo P., Sponta A., Coppolino M.F. (2012). Clinical pharmacology of non-steroidal anti-inflammatory drugs: A review. Antiinflamm. Antiallergy Agents Med. Chem..

[11] Gudeman S.D., Eisele S.A., Heidt R.S., Colosimo A.J., Stroupe A.L. (1997). Treatment of plantar fasciitis by iontophoresis of 0.4% dexamethasone. A randomized, double-blind, placebo-controlled study. Am. J. Sports Med..

[12] Osborne H.R., Allison G.T. (2006). Treatment of plantar fasciitis by LowDye taping and iontophoresis: Short term results of a double blinded, randomised, placebo controlled clinical trial of dexamethasone and acetic acid. Br. J. Sports Med..

[13] Dick I.P., Scott R.C. (1992). Pig ear skin as an in-vitro model for human skin permeability. J. Pharm. Pharmacol..

[14] Jacobi U., Kaiser M., Toll R., Mangelsdorf S., Audring H., Otberg N., Sterry W., Lademann J. (2007). Porcine ear skin: An in vitro model for human skin. Skin Res. Technol..

[15] Tyagi V., Del Río-Sancho S., Lapteva M., Kalia Y.N. (2019). Topical iontophoresis of buflomedil hydrochloride increases drug bioavailability in the mucosa: A targeted approach to treat oral submucous fibrosis. Int. J. Pharm..

[16] Brennan T.J., Vandermeulen E.P., Gebhart G.F. (1996). Characterization of a rat model of incisional pain. Pain.

[17] Zhu Q., Sun Y., Mao L., Liu C., Jiang B., Zhang W., Li J. (2016). Antinociceptive effects of sinomenine in a rat model of postoperative pain. Br. J. Pharmacol..

[18] Liu X.J., Zhang F.X., Liu H., Li K.C., Lu Y.J., Wu Q.F., Li J.Y., Wang B., Wang Q., Lin L.B. (2012). Activin C expressed in nociceptive afferent neurons is required for suppressing inflammatory pain. Brain.

[19] Qiu C.S., Wyhe L.V., Sasaki M., Sakai R., Swanson G.T., Gereau R.W. (2011). Antinociceptive effects of MSVIII-19, a functional antagonist of the GluK1 kainate receptor. Pain.

[20] Kalaria D.R., Patel P., Patravale V., Kalia Y.N. (2012). Comparison of the cutaneous iontophoretic delivery of rasagiline and selegiline across porcine and human skin in vitro. Int. J. Pharm..

[21] Chen Y., Alberti I., Kalia Y.N. (2016). Topical iontophoretic delivery of ionizable, biolabile aciclovir prodrugs: A rational approach to improve cutaneous bioavailability. Eur. J. Pharm. Biopharm..

[22] Walter K., Kurz H. (1988). Binding of drugs to human skin: Influencing factors and the role of tissue lipids. J. Pharm. Pharmacol..

[23] Hilip A.K., Dubey R.K., Pathak K. (2008). Optimizing delivery of flurbiprofen to the colon using a targeted prodrug approach. J. Pharm. Pharmacol..

[24] Lampe M.A., Burlingame A.L., Whitney J.A., Williams M.L., Elias P.M. (1983). Human stratum corneum lipids: Characterization and regional variations. J. Lipid Res..

[25] Belhan O., Kaya M., Gurger M. (2019). The thickness of heel fat-pad in patients with plantar fasciitis. Acta Orthop. Traumatol. Turc..

[26] Molligan J., Schon L., Zhang Z. (2013). A stereologic study of the plantar fat pad in young and aged rats. J. Anat..

[27] Wible J.H., Barrett T., Devarakonda K., Giuliani M. (2014). Biodistribution of diclofenac following repeated topical applications of two diclofenac sodium formulations to minipigs. Biopharm. Drug Dispos..

[28] Aarons L., Khan A.Z., Grennan D.M., Alam-Siddiqi M. (1986). The binding of flurbiprofen to plasma proteins. J. Pharm. Pharmacol..

[29] U.S. Food and Drug Administration Ansaid® Flurbiprofen Tablets Label. https://www.accessdata.fda.gov/drugsatfda_docs/label/2006/018766s013lbl.pdf.

[30] Jamali F., Berry B.W., Wright M.R. (1994). Dose-dependency of flurbiprofen enantiomer pharmacokinetics in the rat. J. Pharm. Sci..

[31] Marnett L.J., Kalgutkar A.S., Pairet M., Ryn J.V. (2004). Structural diversity of selective COX-2 inhibitors. COX-2 Inhibitors.

[32] Santini G., Sciulli M.G., Panara M.R., Padovano R., di Giamberardino M., Rotondo M.T., Soldato P.D., Patrignani P. (1996). Effects of flurbiprofen and flurbinitroxybutylester on prostaglandin endoperoxide synthases. Eur. J. Pharmacol..

[33] Schaible H.G., Ebersberger A., Natura G. (2011). Update on peripheral mechanisms of pain: Beyond prostaglandins and cytokines. Arthritis Res. Ther..

[34] Muraoka A., Tokumura T., Machida Y. (2004). Evaluation of the bioavailability of flurbiprofen and its beta-cyclodextrin inclusion complex in four different doses upon oral administration to rats. Eur. J. Pharm. Biopharm..

